# Etiology and prognosis of nephrocalcinosis according to gestational age in Korean children

**DOI:** 10.1186/s12887-023-04293-7

**Published:** 2023-09-08

**Authors:** Jinwoon Joung, Heeyeon Cho

**Affiliations:** Department of Pediatrics, Samsung Medical Center, Sungkyunkwan University School of Medicine, 81 Irwon-ro, Gangnam-gu, Seoul, 06351 Korea

**Keywords:** Nephrocalcinosis, Preterm birth, Genetics

## Abstract

**Background:**

Nephrocalcinosis (NC) is defined as deposition of calcium in renal tubules and interstitium and is highly related with prematurity and monogenic diseases. Recent studies have reported that NC might be a specific finding of underlying hereditary renal diseases. This study evaluated the risk factors, underlying monogenic causes, and clinical outcomes of NC in Korean children according to gestational age (GA).

**Methods:**

A total of 464 patients younger than 18 years who were diagnosed with NC by ultrasonography from January 2013 to December 2022 in Samsung Medical Center were enrolled. Medical record data of sex, GA, birth weight, underlying disease, medication history, ultrasonography and genetic analysis were reviewed retrospectively.

**Results:**

The male to female ratio was 1:0.98, and the mean age at first diagnosis of NC was 385 days. Approximately 62% of patients experienced confirmed resolution of NC after about one year. In comparison of the preterm (mean GA 28 weeks and 2 days) and full-term (mean GA 38 weeks and 2 days) groups, bronchopulmonary dysplasia, patent ductus arteriosus, and use of furosemide and vitamin D were more frequent in the preterm group. In the full-term group, a larger proportion of cases showed persistent NC without resolution and chronic kidney disease (CKD). Genetic analyses were performed in 56 patients, and the monogenic mutation rate was significantly higher in full-term children (OR 10.02, 95% CI [2.464–40.786], p = 0.001).

**Conclusion:**

While the overall outcomes of pediatric NC are favorable, underlying monogenic causes should be studied, especially in full-term patients without known clinical risk factors.

## Background

Nephrocalcinosis (NC) refers to deposition of calcium in renal tubules and interstitium and differs from nephrolithiasis (NL), which is solid stone formation in the kidney. Both NC and NL are highly associated with morbidities of rare tubulopathies and chronic kidney disease (CKD) [1–3]. The incidence of NC and NL in children has been increasing over recent decades, although the rate varies depending on geographic, socioeconomic, and genetic backgrounds [2–4]. Up to 40% of children are diagnosed with NC or NL incidentally, suggesting that the prevalence is underestimated [3, 4]. NC has been suggested to be caused by factors such as preterm birth, metabolic disorders, and monogenic diseases [3–5]. However, the etiology of NC and NL is not fully established.

Prematurity is one of the leading causes of pediatric NC due to kidney immaturity at birth, especially of the tubules, and exogenous factors after birth [5]. While the exact incidence rate of NC is unknown, the prevalence of NC in preterm neonates is 7–41% [5, 6]. The rate is higher in subjects with gestational age (GA) < 32 weeks or birth weight < 1,500 g [5–7]. Factors such as furosemide and glucocorticoid use, calcium and vitamin D supplementation, parenteral nutrition, and respiratory or circulatory conditions causing acidosis are closely related with the pathogenesis of NC [5–7].

Monogenic mutations were recently identified as a substantial cause of NC. Halbritter et al. [8] and Braun et al. [9] reported that 20.8% and 16.8%, respectively, of early-onset NC and NL cases harbored causative monogenic mutations. Daga et al. [10] revealed 29.4% of total mutations were monogenic by whole exome sequencing (WES) in a study of 51 families with NC and NL. The mutation detection rate was higher in patients with earlier onset and positive family history [10].

We hypothesized that etiology and prognosis of NC may depend on GA (preterm versus full-term births). In the current study, we evaluated and compared the risk factors, underlying monogenic causes, and clinical outcomes of Korean children with NC according to GA.

## Methods

### Study population

A total of 464 patients younger than 18 years who were diagnosed NC with ultrasonography from January 2013 to December 2022 in Samsung Medical Center were enrolled. Patients without (1) available GA or (2) definite diagnosis of NC on sonographic reading were excluded. Data from medical records including sex, GA, birth weight, underlying diseases, birth and medication history, ultrasonography and genetic analysis were retrospectively reviewed. Genetic analysis included targeted gene panel, WES, and whole genome sequencing (WGS) to detect monogenic mutations.

### Definitions

Nephrocalcinosis was diagnosed by ultrasonography. On ultrasound examination, multifocal scattered echogenic foci at the medulla or a diffuse hyperechoic medulla was diagnosed as medullary nephrocalcinosis [11, 12]. Focal bright hypergenic foci with posterior shadowing or gravity dependency at the collecting system were regarded as nephrolithiasis. We enrolled patients with sonographic readings of ‘nephrocalcinosis’ or ‘medullary nephrocalcinosis’ by radiologists and excluded patients with solitary nephrolithiasis.

Preterm birth was defined as birth before 37 weeks of pregnancy. Bronchopulmonary dysplasia (BPD) was diagnosed based on the consensus of the National Institute of Child Health and Human Development (NICHD) criteria [13], and patent ductus arteriosus (PDA) was positive only for patients in which medical or surgical interventions were performed. Vesicoureteral reflux (VUR) was diagnosed by voiding cystourethrography (VCUG). CKD was classified according to the 2012 Kidney Disease: Improving Global Outcomes (KDIGO) guideline [14].

### Statistical analysis

Statistical analysis was performed using SPSS software (version 25.0; SPSS Inc., Chicago, IL, USA). Student’s *t*-test and Chi square test were used for comparing preterm and full-term birth groups. Logistic regression analysis was conducted to estimate and compare clinical risks of NC. *P*-value < 0.05 was considered significant.

## Results

### Demographic and clinical data

The sex ratio in the patient group showed an even distribution as 1:0.98. (Table 1). The number of patients born premature was three-fold higher than that of patients with full-term birth. History of BPD and use of furosemide, vitamin D, and steroids were relatively frequent among the patients since a large proportion of them were premature. Fifteen patients were diagnosed with vesicoureteral reflux (VUR) associated with NC due to urinary stasis and frequent urinary tract infection (UTI), increasing the risk of crystallization [15, 16].

**Table 1:** Demographic and clinical data

Characteristics	Value
Male : Female, n (ratio)	234:230 (1:0.98)
Preterm : Full-term birth, n (ratio)	349:115 (3:1)
Gestational age, mean (weeks + days)	31 + 2
Birth weight, mean ± SD (g)	1699.5 ± 1014.7
Underlying diseases	
Maternal oligohydramnios, *n* (%)	15 (3.2)
BPD, *n* (%)	190 (40.9)
PDA, *n* (%)	135 (29.1)
VUR, *n* (%)	15 (3.2)
Medication history	
Furosemide, *n* (%)	279 (60.1)
Vitamin D, *n* (%)	221 (47.6)
Steroids^a^, *n* (%)	242 (52.2)
Age at first diagnosis of NC, mean ± SD (d)	385.4 ± 875.1
Clinical outcomes	
Persistent follow-up, n (%)	275 (59.3)
Resolution of NC, *n* (%)	171 (62.2)
Sustained NC, *n* (%)	75 (27.3)
Expired, *n* (%)	29 (10.5)
Loss of follow-up, *n* (%)	189 (40.7)
Progression to CKD, *n* (%)	23 (5.0)

*SD* standard deviation; *BPD* bronchopulmonary dysplasia; *PDA* patent ductus arteriosus; *VUR*; vesicoureteral reflux; *NC* nephrocalcinosis; d days; *CKD* chronic kidney disease

^a^Steroids include prednisolone, methylprednisolone, dexamethasone, and hydrocortisone

The mean age at first diagnosis of NC was 385.4 days. Approximately 40% (189/464) of the total patients were lost to follow-up. Among the 275 patients with persistent follow-ups, 29 (10.5%) patients expired without noted improvement of NC, and except them,171 (62.2%) showed confirmed resolution of NC, while 75 (27.3%) sustained NC. Twenty-three patients with NC progressed to CKD (Table 1).

### Comparison of clinical factors associated with NC according to GA

The male-to-female ratio was close to 1:1 in both the preterm and full-term birth groups (Table 2). The mean GA was 28 weeks and 2 days in the preterm group and 38 weeks and 2 days in the full-term group, and the mean birth weight was 1257.7 g and 3039.9 g, respectively. Morbidity of BPD and PDA was frequent in the preterm group, as was furosemide and vitamin D use. The prevalence of VUR was relatively higher in the full-term birth group.

**Table 2:** Comparison of clinical data and outcomes of nephrocalcinosis according to gestational age

Characteristics	Preterm birth (*n* = 349)	Full-term birth (*n* = 115)	*p*-value
Male : Female, n (ratio)	175:174 (1:0.99)	59:56 (1:0.94)	0.850
Gestational age, mean (weeks + days)	28 + 2	38 + 2	< 0.001*
Birth weight, mean ± SD (g)	1257.7 ± 692.2	3039.9 ± 556.4	< 0.001*
Underlying diseases			
Maternal oligohydramnios, *n* (%)	13 (3.7)	2 (1.7)	0.296
BPD, *n* (%)	186 (53.3)	4 (3.5)	< 0.001*
PDA, *n* (%)	122 (35.0)	13 (11.3)	< 0.001*
VUR, *n* (%)	6 (1.7)	9 (7.8)	0.001*
Medication history			
Furosemide, *n* (%)	225 (64.5)	54 (47.0)	0.001*
Vitamin D, *n* (%)	204 (58.5)	17 (14.8)	< 0.001*
Steroids^a^, *n* (%)	183 (52.4)	59 (51.3)	0.833
Age at first diagnosis of NC, mean ± SD (d)	229.6 ± 587.8	858.0 ± 1325.0	< 0.001*
Duration to resolution, mean (d)	427.4 ± 620.9	430.4 ± 374.4	0.976
Clinical outcomes			
Persistent follow-up, n (%)	187 (53.6)	88 (76.5)	
Resolution of NC, *n* (%)	128 (68.4)	43 (48.9)	0.003*
Sustained NC, *n* (%)	42 (22.5)	33 (37.5)	
Expired, *n* (%)	17 (9.1)	12 (13.6)	0.033*
Loss of follow-up, *n* (%)	162 (46.4)	27 (23.5)	0.001*
Progression to CKD, *n* (%)	11 (3.2)	12 (10.4)	0.002*

*SD* standard deviation, *BPD* bronchopulmonary dysplasia, *PDA* patent ductus arteriosus, *VUR* vesicoureteral reflux, *NC* nephrocalcinosis, *d* days, *CKD* chronic kidney disease

^a^Steroids include prednisolone, methylprednisolone, dexamethasone, and hydrocortisone

NC was first diagnosed at a mean age of 229.6 days and 858.0 days in the preterm and full-term groups, respectively. Clinical outcomes of NC were significantly different between groups. Excluding those who were lost to follow-up, the rate of spontaneous resolution was significantly higher in the preterm group (68.4% vs. 48.9%, p = 0.003). A larger proportion of patients from the full-term group experienced with persistent NC compared with preterm patients (OR 2.34, 95% CI [1.320–4.144], p = 0.004). In cases where NC has improved, the mean duration to resolution from first diagnosis was approximately 14 months regardless of GA (preterm 427 days vs. full-term 430 days). The mortality rate was higher in the full-term group, while loss of course following NC was more frequent in the preterm group. Regarding renal function, CKD was significantly more prevalent in full-term patients (OR 3.58, 95% CI [1.534–8.354], p = 0.003).

### Genetic analysis and detected monogenic mutations

Gene studies were performed in 56 of the 464 patients (preterm 8.6% vs. full-term 22.6%, p < 0.001) (Table 3). The total detection rate of monogenic mutations was 64.3% and was significantly higher (88.5%) in full-term patients (OR 10.02, 95% CI [2.464–40.786], p = 0.001).

**Table 3:** Genetic analysis and the detection rate of mutations

Characteristics	Preterm birth (*n* = 349)	Full-term birth (*n* = 115)	*p*-value
Genetic analysis^a^, *n* (%)	30 (8.6)	26 (22.6)	< 0.001*
Detection rate, *n* (%)	13/30 (43.3)	23/26 (88.5)	< 0.001*

^a^Genetic analysis include gene panels and exome/genome sequencing

Monogenic mutations were detected in 32 genes from 36 individuals (Table 4). *PKD1*, *ALPL*, and *KMT2D* mutations were discovered in more than one patient. Five of the mutated genes (*ALPL*, *CDKN1C*, *CLCNKB*, *HPRT1*, and *OCRL*) were listed in the 83 reported genes causing NC as defined by the Online Mendelian Inheritance in Man (OMIM) [15]. Genes identified in patients who developed CKD included *CFH*, *HNF1β*, *HPRT1*, and *PAX2*. As genetic analysis was mostly presented for evaluation of anomalies and was not targeted for NC, numerous genetic diagnoses were related with neuromuscular disorders or enzyme deficiencies.

**Table 4:** Genetic mutations detected in 464 pediatric patients with nephrocalcinosis

No.	Gene	Nucleotide change	Amino acid change	Zygosity state	Term vs. Preterm birth	Diagnosis
1	*ACTA1*	c.863 A > T	p.Asp288Val	hetero	preterm	Nemaline myopathy
2	*ADNP*	c.2358dup	N/A	hetero	term	Helsmoortel-van der As syndrome
3	*ALPL* ^a^	c.334G > A c.1039 C > T	p.Gly112Ser p.Gln347*	hetero hetero	term	Infantile hypophosphatemia
		c.1052 A > G c.1559delT	p.Glu351Gly p.Leu520Argfs*86	hetero hetero	term	Infantile hypophosphatemia
4	*CDKN1C* ^a^	c.821-9 C > A	-	N/A	preterm	Beckwith-Wiedemann syndrome
5	*CFH* ^b)^	c.1064 A > C c.184G > A	p.Tyr355Ser p.Val62Ile	hetero hetero	term	Atypical hemolytic uremic syndrome (aHUS)
6	*CLCNKB* ^a)^	c.139G > A	p.Gly465Glu	hetero	term	Bartter syndrome, type 3
7	*CNTNAP1*	c.2901_2902del	p.Cys968Phefs*11	homo	preterm	Congenital hypomyelinating neuropathy
8	*COQ8B*	c.737G > A	p.Ser246Asn	hetero	term	Coenzyme Q10 deficiency
9	*CREBBP*	c.14dup	p.Leu5Phefs*22	hetero	term	Rubinstein-Taybi syndrome
10	*FZD4*	c.205 C > T	p.His69Tyr	N/A	term	Familial exudative vitreoretinopathy
11	*GAA*	c.875 A > G c.1822 C > T	p.Tyr292Cys p.Arg608*	comp comp	term	Glycogen storage disease, type 2 (Pompe disease)
12	*GBA*	c.754T > A c.887G > A	p.Phe252Ile p.Arg296Gln	hetero hetero	term	Gaucher disease
13	*GNA11*	c.548G > C	p.Arg183Pro	hetero	preterm	Hypocalciuric hypercalcemia, type 2
14	*HNF1β* ^b^	N/A	N/A	N/A	term	ADTKD-HNF1β
15	*HPRT1* ^a,b^	c.151 C > T	p.Arg51Ter	hemi	term	Lesch-Nyhan syndrome
16	*IDS*	c.851 C > T	p.Pro284Leu	hemi	term	Mucopolysaccharidosis, type 2
17	*IL2RG*	c.548T > C	p.Leu183Ser	hemi	preterm	X-linked SCID
18	*KMT2D*	c.3103 C > t	p.Gln1035*	hetero	preterm	Kabuki syndrome
		c.5268delG	p.Arg1757Glufs*28	N/A	term	Kabuki syndrome
19	*KRT14*	c.377T > A	p.Leu126Gln	hetero	term	Inherited epidermolysis bullosa simplex
20	*MMP13*	c.212T > C	p.Met71Thr	hetero	preterm	Spondyloepimetaphyseal dysplasia (SEMD)
21	*MTOR*	c.7501 A > T	p.Ile2501Phe	N/A	preterm	West syndrome
22	*MUT*	c.1105 C > T c.2131G > T	p.Arg369Cys p.Glu711*	hetero hetero	preterm	Methylmalonic acidemia
23	*OCRL* ^a^	c.739T > A	p.Trp247Arg	hemi	preterm	Lowe syndrome
24	*OTC*	c.780_781ins	p.Ile261Glnfs*35	N/A	term	OTC deficiency
25	*PAX2* ^b^	c.69delinsCG	p.Val26Cysfs*3	hetero	preterm	Papillo-renal syndrome
26	*PHOX2B*	Polyalanine repeat mutation (20/26)			term	Central hypoventilation syndrome
27	*PKD1*	c.4609G > T	p.Glu1537*	hetero	preterm	ADPKD
		c.9355 A > T	p.Lys3119Ter	hetero	term	ADPKD
		N/A	N/A	hetero	term	ADPKD
28	*PKHD*	c.6840G > A	p.Trp2280*	N/A	preterm	ARPKD
29	*SCN2A*	N/A	N/A	N/A	term	Early infantile epileptic encephalopathy (EIEE)
30	*SLC25A13*	c.852_855del c.1180 + 1G > A	p.Met285Profs*2 -	hetero homo	term	Citrullinemia, type 2
31	*SLC37A4*	c.149G > A c.1042_1043del	p.Gly50Glu p.Leu348Valfs*53	comp comp	term	Glycogen storage disease, type 1B
32	*VPS33B*	c.403 + 2T > A	-	homo	term	ARC syndrome

*N/A* not available, *hetero* heterozygous, *homo* homozygous, *comp* compound heterozygous, *hemi* hemizygous, *ADTKD* autosomal dominant tubulointerstitial kidney disease, *SCID* severe combined immunodeficiency, *OTC* ornithine transcarbamylase, *ADPKD* autosomal dominant polycystic kidney disease, *ARPKD* autosomal recessive polycystic kidney disease, *ARC* arthrogryposis, renal dysfunction and cholestasis

^a^ previously reported causative genes of nephrocalcinosis

^b^ gene detected in patient who developed chronic kidney disease (CKD)

## Discussion

In this study, NC was more prevalent in the preterm group, and prematurity and use of furosemide, vitamin D, and steroids were clinical risk factors of NC [5–7]. Recognized risk factors of NC and routine screening of ultrasonography during hospitalization may be contributing factors of the incidence. The long-term outcome of NC in preterm patients was generally favorable, showing a high rate of spontaneous resolution, in agreement with previous studies, whereas full-term children with NC were at significant risk of persistent NC and of CKD compared with preterm patients [5, 16–18]. The proportion of lost patients in this study group, nearly 50%, is considerable. While spontaneous resolution is noted in most pediatric NC cases, NC in preterm neonates has been reported to increase the risk of long-term renal complications such as stunted kidney growth, increased blood pressure, and altered tubular or glomerular functions [5, 19–23].

The total mutation detection rate in this cohort was 64.3%, which was higher than previous reports of 16.7–29.4% [8–10]. In 2015, Halbritter et al. [8] reported that causative monogenic mutations of NC were detected more frequently in pediatric patients (defined by age of onset < 18 years) compared with the adult group (20.8% vs. 11.4%). In 2018, Daga et al. [10] reported that all molecular genetic diagnoses related to NC had occurred in patients younger than 15 years. Presence of family history was also suggested to correlate with a higher mutation detection rate [10]. In our results, underlying monogenic causes were more prevalent in the full-term group. Only 15% of mutated genes identified in this study (5/32 genes) were defined as causative genes of NC by OMIM. However, several genes specific for hereditary or genetic kidney disease were detected including *CFH, COQ8B, HNF1β, PAX2, PKD1*, and *PKHD*. Most other genes also likely contributed to NC, considering the pathophysiology of derived disease. There were additional three monogenic causes discovered in full-term patients who were excluded due to unclear GA. Mutations in *CLCN5, PHEX*, and *NPHP4* which are listed in the gene list in OMIM, were detected in the three patients. Molecular genetic diagnoses have clinical implications for NC patients. Confirmed genetic information can help guide clinicians to monitor individuals and provide precise, timely treatment. Since the phenotypes of each genetic disease are heterogeneous, it is important to determine appropriate candidates and the specified type of gene study. Our results from the current cohort revealed that NC in full-term patients without typical clinical risk factors had a higher prevalence of causative monogenic mutations. Accordingly, we recommend mutational analysis for (1) patients with early-onset NC before age of 15 years with or without family history and (2) full-term born children without clinical risk factors of NC.

There is lack of consensus regarding the efficacy or superiority for genetic analysis for NC. However, as unknown monogenic causes being constantly discovered from studies, gene panels may not provide optimal coverage [8–10, 17]. The known monogenic causes defined by OMIM also have been actively updated, and the list increased to 83 from 30 genes in 2010. Therefore, WES may be an advantageous tool distinguishing heterogeneous genetic conditions presenting NC until the etiology and phenotype-genotype correlation are sufficiently established. Further, WES may be helpful for physicians to arrange personalized treatment plans in the early stage of genetic diseases or syndromes with no characteristic symptoms.

To the best of our knowledge, this is the first study to compare the outcomes of NC and demonstrate the clinical outline of NC in accordance with gestational age. However, since the current study was a retrospective review, there are a few limitations. First, there was no standardized type of genetic analysis since the original purpose of genetic analysis was not only for NC itself or renal diseases but also other hereditary or genetic diseases. This is a limitation of retrospective study. As all the patients enrolled in this cohort necessarily carry NC which is a possible renal manifestation of various genetic diseases, there is a clinical comparability between groups of different GA presenting NC with or without other anomalies. Second, as the current cohort was from tertiary center, severity of underlying diseases could possibly affect the clinical characteristics of enrolled patients including medication history. Third,, there was a lack of clinical information. Several patients with valid genetic disorders or secondary tubulopathies induced by hemato-oncologic diseases were excluded due to unclear GA. Some patients with monogenic mutation did not have available detailed genetic information since they underwent WES/WGS in another center. Last, only glomerular function was used to represent renal outcomes. Other findings suggesting tubular dysfunction and hypertension were not discussed. Further research on the genotype-phenotype correlation of NC and precise indications for gene study are expected along with long-term renal outcomes.

In conclusion, the current study indicates that persistent NC and progression to CKD in a notable proportion of our cohort, which raises the possibility of comorbidities including hypercalciuria, nephrolithiasis, and other CKD-related complications [21–23]. Additionally, underlying monogenic causes contribute to a substantial proportion in full-term born children. Therefore, we emphasize the necessity of (1) assessing underlying monogenic causes with WES in patients with early onset NC, especially in full-term born patients without known clinical risk factors, and (2) continuous monitoring for patients with NC regardless of GA.

## Data Availability

The datasets used and/or analyzed during the current study are available from the corresponding author on reasonable request.
